# Discrimination of wear and non-wear in infants using data from hip- and ankle-worn devices

**DOI:** 10.1371/journal.pone.0240604

**Published:** 2020-11-02

**Authors:** Samuel R. LaMunion, Scott E. Crouter, Nicholas T. Broskey, Abby D. Altazan, Leanne M. Redman

**Affiliations:** 1 Department of Kinesiology, Recreation, and Sport Studies, The University of Tennessee, Knoxville, TN, United States of America; 2 Reproductive Endocrinology and Women’s Health Laboratory, Pennington Biomedical Research Center, Baton Rouge, LA, United States of America; University of Pittsburgh, UNITED STATES

## Abstract

**Introduction:**

A key component to analyzing wearable sensor data is identifying periods of non-wear. Traditionally, strings of consecutive zero counts (e.g. >60-minutes) are identified indicating periods of non-movement. The non-movement window length is then evaluated as wear or non-wear. Given that non-movement is not equivalent to non-wear, additional criteria should be evaluated to objectively identify periods of non-wear. Identifying non-wear is especially challenging in infants due to their sporadic movement, sleep frequency, and proportion of caregiver-generated movement.

**Purpose:**

To use hip- and ankle-worn ActiGraph wGT3X-BT (wGT3X-BT) data to identify non-wear in infants.

**Methods:**

Fifteen infant participants [mean±SD; age, 8.7±1.7 weeks (range 5.4–11.3 weeks); 5.1±0.8 kg; 56.2±2.1 cm; n = 8 females] wore a wGT3X-BT on the hip and ankle. Criterion data were collected during two, 2-hour directly observed periods in the laboratory. Using raw 30 Hz acceleration data, a vector magnitude and the inclination angle of each individual axis were calculated before being averaged into 1-minute windows. Three decision tree models were developed using data from 1) hip only, 2) ankle only, and 3) hip and ankle combined.

**Results:**

The hip model classified 86.6% of all minutes (wear and non-wear) correctly (F1 = 75.5%) compared to the ankle model which classified 90.6% of all minutes correctly (F1 = 83.0%). The combined site model performed similarly to the ankle model and correctly classified 90.0% of all minutes (F1 = 80.8%).

**Conclusion:**

The similar performance between the ankle only model and the combined site model likely indicates that the features from the ankle device are more important for identifying non-wear in infants. Overall, this approach provides an advancement in the identification of device wear status using wearable sensor data in infants.

## Introduction

There is a growing interest in tracking physical activity (PA) throughout the lifespan, starting during infancy [1], to evaluate the relationship with PA and childhood obesity among other chronic disease conditions known to have early onset as well as to describe movement patterns and PA levels as infants develop [2]. Descriptions of infant movement and monitoring PA early in life, as a marker of development status and in relation to maternal PA [3, 4] is rapidly expanding [5, 6]. Evaluating infant PA is complicated. Infant movement is frequent, intermittent, and may also be largely influenced by a caregiver such as when the infant is sleeping or waking and being held or carried. One method that can be used to objectively capture infant movement is accelerometer-based physical activity (PA) monitors and other wearable sensors. These devices are being developed to objectively capture infant movement patterns including PA behaviours. These small, non-invasive, lightweight devices have been routinely used as an objective method to assess free-living PA in other populations and are also suitable for use in infants to begin the evaluation of developing movement patterns [7–11].

One challenge of working with PA monitor data, in general, is identifying device wear time and non-wear time during post-processing. This is a necessary step so that monitor data can be correctly aggregated and summarized so that the PA metrics calculated are reflective of only the time an individual is wearing the device. It can, however, be challenging to identify periods of wear and non-wear without additional information such as wear time logs. The main contributor to this issue is the identification of true device non-wear time [12]. Frequently, extended periods of non-movement are misidentified as periods of non-wear [12]. Presently, most non-wear methods have been developed for use with hip-worn monitor data using activity counts, a proprietary metric used to quantify activity that is based on filtered and integrated raw acceleration data in a given window of time (e.g. 60 seconds) [13–17]. The activity count value is intended to represent the movement that occurs during a specified window length with a zero-count value representing no movement. To determine non-wear time, strings of consecutive zero counts are identified with the intuition being that extended periods of consecutive zero counts are occurring due to the device not moving and therefore most likely not being worn [13, 14, 17]. Once the strings of consecutive zero counts have been identified, the length of the strings are evaluated and classified as wear or non-wear based on the length of the string of consecutive zero counts. Typically, longer strings of consecutive zero counts are assumed to correspond to periods of non-wear. The window length used to define an extended period of non-movement as non-wear is often tailored to the population (e.g. youth versus adults). These methods rely solely on changes in movement for detection and identification of periods of movement which can be ineffective, especially during extended periods of sedentary behavior (SB) where devices are frequently worn but extended periods of non-movement occur.

Similar methods to those outlined above can be applied to infant PA monitor data, however, as previously mentioned, the large proportion of sleeping time and caregiver generated movement during waking time makes the case with infants more challenging. To date, two studies have examined extracting waking wear time in infants, and both have used ActiGraph activity counts and not raw acceleration data [18, 19]. Ketcheson et al. [18] developed a protocol for the identification of non-wear periods, periods of sleep, and periods containing movement generated by a caregiver resulting in only minutes of wear time where the infant was awake. These identified periods were cleaned from the dataset based on parent logs along with any periods of two or more minutes of consecutive zero count values leaving only waking wear time remaining. Similarly, Pitchford et al. [19] removed data as non-wear from any periods containing two or more minutes of consecutive zero counts as this was considered representative of non-waking time. The issue with these approaches is that the success of the protocol is contingent upon manual review based on matching parental logs and relying on short periods of non-movement as non-wear. The intention and motivation for identifying waking wear time is distinctly different from that of simply identifying wear time and therefore the two outcomes cannot be directly compared for that specific purpose. In a head-to-head comparison of the two methods, they should not be expected to perform comparably as the desired outcome is different. However, it is important to note the differences between methods. This work seeks to highlight the shortcoming of using accelerometer counts to quantify infant movement, including the identification of wear time, which is one important aspect of processing infant accelerometer data.

With scarce literature existing on PA monitoring in infants and the limitations of current approaches in youth and adult populations using count data, a new method is needed. To robustly characterize PA and discriminate device wear time, we hypothesize shifting to raw acceleration data from counts and using additional variables beyond device acceleration (i.e. device inclination). Device inclination has previously been used to identify periods of sleep by detecting changes in arm position for wrist-worn devices [20]. Therefore, assessing changes in device inclination and movement should allow for more robust detection of non-wear since the inclination information is supplementary to the device acceleration. Thus, the purpose of this study was two-fold. The primary purpose of the study was to use device inclination paired with device acceleration data from hip- and ankle-worn ActiGraph wGT3X-BT to identify periods of device wear and non-wear in infants. Secondarily, the purpose of this study was to compare a novel decision tree method for identifying wear and non-wear using raw acceleration data to a previously developed method designed to identify waking wear time using count data.

## Methods

### Participants

This is a cross-sectional analysis undertaken as part of an observational study designed to develop robust methods for measuring energy metabolism and physical activity in infants. The study was approved by the Pennington Biomedical Institutional Review Board and was registered as a clinical trial named Measurement of Energy Metabolism in Infants (BabyEE Pilot; NCT02683473). Parents of 15 healthy, full-term infants from Baton Rouge, LA, and the surrounding areas were recruited through social media advertisements, targeted emails, flyers, and word of mouth. Initial eligibility criteria were assessed through an online screening instrument. Healthy, full-term infants aged between 1 and 3 months with no evidence of congenital abnormality or gastric reflux and no use of medications to treat chronic conditions were invited to participate. Written informed consent was obtained from both parents or guardians prior to the initiation of procedures at the first visit. A data transfer protocol was established, and Institutional Review Board approval was obtained at the University of Tennessee, Knoxville for data analysis.

### Procedures

Infants completed two visits at the research center approximately 6–7 days apart. During the first laboratory visit, body weight was measured on a calibrated scale with the infant unclothed (SCALE-TRONIX, White Plains, NY). Length was measured twice with the infant's head in the Frankfort position using an infantometer with a stationary headboard and a moveable footboard. Fat mass and fat-free mass were assessed using air displacement plethysmography (PEA POD, COSMED, Concord, CA) with the infant wearing a head cap to cover the hair.

At the initial visit, infants were fitted with two ActiGraph wGT3X-BT devices, which were used to assess infant movement in free-living conditions between study visits. The wGT3X-BT is a small (3.3 x 4.6 x 1.5 cm), lightweight (19 grams), triaxial accelerometer (dynamic range ±8 *g*) that is frequently used in research studies [21]. One device was attached to the right or left hip by Velcro securely fixed on infant-sized underpants that were worn over the diaper with the USB cap pointing up [i.e. vertical axis (y) -1*g* if the infant were to be placed in standard anatomical position]. A second monitor was attached to the right or left ankle proximal to the lateral malleolus with the USB cap pointing up the leg [i.e. vertical axis (y) -1g if the infant were to be placed in standard anatomical position] by a Velcro ActiGraph strap. Parents were instructed to leave both monitors on the infant and only to remove them if necessary, such as during clothes changing or bathing.

On the first and last day of data collection, participants were observed in the laboratory for approximately 2-hours. Infants were directly observed by a member of the research team throughout their laboratory visits. During this time, infants were placed either supine on an activity mat, reclining in an infant rocking chair, or held by a caregiver and directly observed to make a record of general behavior during the assessment. Normal infant activity was not manipulated or scheduled but was recorded by a member of the research team. Habitual activities noted were sleeping, feeding, crying, bouncing of the rocker, diaper changing, being swaddled, and being held by caregivers or members of the research team with annotations being made to the nearest minute. Infants spent the majority (72.8%) of the laboratory visit in the infant rocking chair. Observer notes were used to create criterion labels for each minute including whether the device was being worn or not and whether the participant was waking or sleeping. All available minutes with observer notes from both laboratory visits were pooled for each participant creating the overall observation time. This time includes both wear and non-wear periods and sleeping and waking time. The origin of the criterion minutes of wear and non-wear is broken down as follows. During the two-hour observation periods, there were no minutes of non-wear after the devices were placed onto the infants. The non-wear time was obtained during two different periods when the non-wear could be determined: 1) the period after initialization and before the participant arrived for the first laboratory visit, and 2) the period after the device was removed following the second laboratory visit until it was downloaded. During this time, the device was in the research laboratory, positioned on a table not being worn or moved, which is similar to how a device would be placed during periods of non-wear in free-living situations. These periods comprised the bulk of the criterion non-wear time used for classifier development (n = 6,771 minutes of non-wear).

### Data reduction and processing

#### Raw acceleration data

Raw triaxial wGT3X-BT data were collected at 30 Hz. During the data reduction and aggregation process, additional variables were calculated from the raw 30 Hz triaxial acceleration data (X-, Y-, and Z-axis acceleration). Additional variables included vector magnitude $\left(VM=\sqrt{Y^{2}+X^{2}+Z^{2}}\right)$, and X-, Y-, and Z-axis inclination angles $\left(ex.ϴY=\cos^{-1}\frac{Y}{\sqrt{Y^{2}+X^{2}+Z^{2}}}=radians\;x\frac{180}{Pi}=axis\;angle\;in\;degrees\right)$. Individual axis acceleration (milli-g), VM (milli-g), and individual axis inclination angle (degrees) data were then averaged into 1-minute windows. Additionally, to indicate a change in movement, a sliding coefficient of variation $\left(CV=(\frac{sample\;standard\;deviation}{sample\;mean})\;x\;100=\%\;relative\;variability\;in\;a\;window\right)$ of X-, Y-, and Z-axis acceleration, and VM was calculated for each 1-minute window of data. To accomplish the calculation of the CV, the preceding minute(s) and succeeding minute(s) were included to create a window for the CV calculation. Windows lengths considered include 2-, 3-, 4-, and 5- minute windows. The CV of each window provides a measure of the relative variability in movement over that specific window length. Specifically, to calculate the CV, the preceding minute(s) and succeeding minute(s) were included to create a window for the CV calculation. Four windows lengths were considered; 2-, 3-, 4-, and 5- minutes. The CV of each window provides a measure of the relative variability in movement over that time period. Using a two-minute window length as an example, a CV was obtained for a single minute by calculating the CV of two consecutive minutes, including the minute of interest as the anchor point. This was done looking backward using the anchor minute and the preceding minute (i.e. the minute occurring just before the minute of interest) and the anchor minute and the succeeding minute (i.e. the minute occurring just after the minute of interest). The minimum of these two CV values was retained as the CV of the anchor minute. An example of the CV calculation is shown in Table 1. These additional variables were selected on the basis of incorporating device inclination and change in movement as predictors of device wear time. The final training dataset is available in the S1 Dataset.

**Table 1:** Sample coefficient of variation (CV) calculation for the two- and three-minute window size.

		Two-Minute CV Calculation		Three-Minute CV Calculation			Final CV Values used in models for each minute	
Timestamp	VM (mg)	CV Two Backwards (%)	CV Two Forwards (%)	CV Three Backwards (%)	CV Three Center (%)	CV Three Forwards (%)	Minimum CV Two (%)	Minimum CV Three (%)
10:30:00	1020.73							
10:31:00	1021.07							
10:32:00	1021.28	1.5	0.1	2.7	1.1	1.1	0.1	1.1
10:33:00	1021.3	0.1	1.2	1.2	1.1	1.8	0.1	1.1
10:34:00	1021.48	1.2	2.5	1.1	1.8	83.7	1.2	1.1
10:35:00	1021.12	2.5	103.5	1.8	83.7	77.0	2.5	1.8
10:36:00	1036.18	103.5	80.6	83.7	77.0	235.8	80.6	77.0
10:37:00	1024.44	80.6	241.5	77.0	235.8	257.6	80.6	77.0
10:38:00	990.04	241.5	113.8	235.8	257.6	109.1	113.8	109.1
10:39:00	974.24							
10:40:00	969.71							

VM = Vector Magnitude; mg = milli-*g*’s.

#### Count data

Raw 30 Hz acceleration data were downloaded and converted to 60-s counts with the low-frequency extension (LFE) enabled using Actilife (version 6.13.3). The 60-s VM counts were used for calculation of wear and non-wear using the Pitchford et al. [19] and Ketcheson et al. [18] consecutive zero counts method.

### Model development

Three decision tree classification models were developed using the *rpart* [22], *rpart*.*plot* [23], and *caret* [24] packages where each minute of collapsed data was classified as “wear” or “non-wear”. Leave-one-out cross-validation (LOOCV) by participant ID was used to assess model performance (i.e. a model was created with data from fourteen of the fifteen participants and tested using the data from the withheld participant. This required the development of fifteen individual trees for each model type (combined attachment site and individual attachment sites) holding each participant out by ID. Model parameters were independently tuned and optimized for each model iteration using the “train” function in the *caret* package. For tuning and optimization, the ‘traincontrol’ argument used k-fold cross-validation with ten folds (e.g. the test data is split into ten subsets, the model is trained on each subset and accuracy is estimated, and finally an overall accuracy estimate is made using the estimated accuracy from each fold). The ‘tunegrid’ argument tried 50 complexity parameter (cp) values between 10^−5^ and 10^−1^. The optimal complexity parameter cp for each model was objectively selected to reduce overfitting by using the ‘best.cp’ value from the trained model using receiver operating characteristics (ROC) analysis. Final models and sample code are available from the corresponding author upon request.

### Statistical analysis

Analyses were performed jointly in *R* statistical software (*R* version 3.4.2 (2017-09-28)–“Short Summer”) [25] and SPSS 24 (IBM Corporation, Armonk, NY). A two-part analysis was conducted: 1) for decision tree model performance using raw acceleration data and 2) a comparison of the decision tree models to the consecutive zero counts approach for identifying wear and non-wear across the entire lab visit and for identifying wear time during periods of sleeping and waking.

#### Decision tree performance

For the purposes of examining model performance, non-wear was treated as the positive class. Individual model classification accuracy (true positives (i.e. non-wear classified as non-wear + true negatives (i.e. wear classified as wear)/(all positives + all negatives), sensitivity (true positives /(true positives + false negatives), and specificity (true negatives/(true negatives + false positives), and F1 Score $\left(2\;x\;(\frac{Sensitivity\;x\;PPV}{Sensitivity+PPV})\right)$, were calculated for each tree and the average of each performance metric is used to summarize total model performance. Performance metrics across all fifteen individual models were averaged to summarize overall model performance for each model type (combined-site, hip only, ankle only) and feature combination. Each model was plotted using the ‘*rpart*.*plot’* [23] package to illustrate the classification process. Model code is available from the corresponding author upon request.

### Consecutive zero counts comparison

Classifications were made for all criterion labelled minutes (i.e. minutes with observer notes from the laboratory visits) by the hip, ankle, and combined site decision trees. The consecutive zero counts method, as described by Pitchford et al. [19] and Ketcheson et al. [18], was applied to 60-s hip and ankle count data independently and also combined so both hip and ankle data were used to make a single classification. The combined decision of wear status for the count data was determined by a single site (e.g. if one site was labelled “wear” then the default group-level decision for the combined site was “wear”). Minutes of wear and non-wear were calculated using each model for comparison of 1) overall time and 2) time spent in the infant rocking chair. The wear time spent in the infant rocking chair was included to examine the estimates of minutes of wear and non-wear with the inclusion of “sleep” and “wake” status of the infant based on observer notes to determine where misclassification might be occurring as a crude way to compare how a method designed for identifying only wear and non-wear compares to a method designed to identify waking wear time.

## Results

The following describes the physical characteristics of the participants [mean±SD; age, 8.7±1.7 weeks (range 5.4–11.3 weeks); weight 5.1±0.8 kg; length 56.2±2.1 cm; n = 8 females]. Model performance summaries are shown in Supplemental Digital Content 1. The final model for each site was selected by evaluating the model with the highest combined classification accuracy and F1 score. This model selection process eliminated all models including raw acceleration data (i.e. X-, Y-, and Z-axis acceleration and VM) for both the hip and ankle attachment sites in favor of relative variability in acceleration (CV). Of the four CV window lengths, the 4-minute CV had the most stable performance between the hip and ankle. This resulted in the final single site and combined site models including individual axis inclination angles (e.g. X-, Y-, and Z-axis) and individual axis (e.g. X-, Y-, and Z-axis) CV values in a 4-minute window. This left six predictors per attachment site for the development of the final single site models: X-, Y-, and Z-axis inclination angles and X-, Y-, and Z-axis 4-minute CV.

Model performance metrics for the final models are presented in Table 2. The single-site ankle model performed best with the highest classification accuracy and F1 score. However, all three models (combined site, hip only, ankle only) performed similarly with ≥86.0% correct classification of device wear and non-wear, sensitivities and specificities ≥77.0% and ≥85.0%, respectively, and F1 scores all ≥75.0%.

**Table 2:** Model performance metrics from a combined site model using hip and ankle data, a hip only model, and an ankle only model.

Site	Model	Accuracy	Sensitivity	Specificity	F1
Hip	Inclination Angles + Individual Axis Acceleration CV-4	86.6%	77.8%	85.6%	75.4%
		(69.5–97.6%)	(0.0–100.0%)	(65.3–100.0%)	(11.0–98.7%)
Ankle	Inclination Angles + Individual Axis Acceleration CV-4	90.6%	86.0%	90.7%	83.0%
		(70.9–99.8%)	(51.5–100.0%)	(72.7–99.7%)	(48.5–99.9%)
Combined	Inclination Angles + Individual Axis Acceleration CV-4	90.0%	88.7%	90.2%	80.8%
		(63.0–99.8%)	(58.2–100.0%)	(57.9–100.0%)	(40.7–99.9%)

Individual Axis Acceleration = X-, Y-, Z-axis acceleration values; Inclination Angles = X-, Y-, and Z- axis inclination angles; CV = Coefficient of variation in acceleration in the specified window of time (i.e. CV-4 is the coefficient of variation in a four-minute window); PPV = Positive Predictive Value, NPV = Negative Predictive Value.

Results of the comparison to the consecutive zero counts method are presented in Tables 3 and 4. The ankle only decision tree model was the closest to criterion non-wear minutes for overall time (0.7%; 50 minutes). Comparatively, the best performing consecutive zero counts model was the ankle for overall time (32.5%, 146.8 minutes). For correctly identifying wear and non-wear during sleeping and waking time, the combined site decision tree model performed best classifying on average 7.1 ± 20.0 minutes as non-wear during sleep time compared to zero criterion non-wear minutes during sleep time. Comparatively, the best performing consecutive zero counts method (combined site) classified on average 88.5 ± 39.3 minutes as non-wear during sleep.

**Table 3:** Summary of the final decision tree models classifications of wear and non-wear and comparison to the consecutive zero counts method.

	Model (Site)	Total^a^ Non-wear Minutes	Total^a^ Wear Minutes	Mean^b^ (SD) Non-wear Minutes	Percent Difference Criterion Non-Wear Minutes	Mean^b^ (SD) Wear Minutes	Percent Difference Criterion Wear Minutes
**Overall**	Criterion	6771	4813	451.4 (725.0)	-	320.9 (131.9)	-
	Ankle Decision Tree	6821	4763	454.7 (764.1)	0.7%	317.5 (115.9)	1.1%
	Combined Decision Tree	6602	4982	440.1 (758.8)	2.5%	332.1 (143.5)	3.5%
	Combined Consecutive Zero Counts	8760	2824	584.0 (778.1)	29.4%	188.3 (85.6)	41.3%
	Ankle Consecutive Zero Counts	8973	2611	598.2 (785.8)	32.5%	174.1 (78.4)	45.7%
	Hip Consecutive Zero Counts	9110	2474	607.3 (781.7)	34.5%	164.9 (80.5)	48.6%
	Hip Decision Tree	4057	7527	270.5 (472.0)	40.1%	501.8 (653.5)	56.4%

^a^Total minutes are a sum of all fifteen participants to demonstrate an overall summary of how many minutes of wear and non-wear each model classified compared to the criterion minutes of wear and non-wear.

^b^Mean minutes of wear and non-wear are the average number of minutes each model classified compared to the criterion minutes.

**Table 4:** Summary of the classifications of wear and non-wear during time spent in an infant rocking chair including sleeping and waking time.

Model (Site)	Total^a^ Non-wear Minutes		Total^a^ Wear Minutes		Mean^b^ (SD) Non-wear Minutes			Mean^b^ (SD) Wear Minutes	
	Sleep	Wake	Sleep	Wake	Sleep	Wake	Sleep		Wake
Criterion	0	0	1987	1519	0.0 (0.0)	0.0 (0.0)	132.4 (61.3)		101.3 (46.1)
Ankle Decision Tree	115	63	1872	1456	7.7 (20.0)	4.2 (13.8)	124.8 (59.4)		97.1 (50.9)
Combined Site Decision Tree	106	82	1881	1437	7.1 (19.9)	5.5 (14.0)	125.4 (62.7)		95.8 (50.3)
Hip Decision Tree	175	37	1812	1482	11.7 (22.5)	2.5 (4.1)	120.8 (50.4)		98.8 (45.9)
Combined Site Consecutive Zeros	1328	206	659	1313	88.5 (39.3)	13.7 (18.0)	43.9 (29.8)		87.5 (53.1)
Ankle Consecutive Zeros	1365	234	622	1285	91.0 (39.6)	15.6 (20.3)	41.5 (29.3)		85.7 (54.1)
Hip Consecutive Zeros	1535	278	452	1241	102.3 (47.1)	18.5 (19.0)	30.1 (20.8)		82.7 (50.9)

^a^Total minutes are a sum of all fifteen participants to demonstrate an overall summary of how many minutes of wear and non-wear each model classified compared to the criterion minutes of wear and non-wear.

^b^Mean minutes of wear and non-wear are the average number of minutes each model classified compared to the criterion minutes.

## Discussion

This study presents a novel method for the identification of device non-wear in infants. To our knowledge, this is the first study to investigate device non-wear in infants using raw sensor data as an objective, standalone method. Other methods exist to score non-wear time using raw sensor data but are incorporated in a larger data reduction process that may not be suitable in all instances [26–28]. Additionally, to our knowledge, this is the first study to use device inclination as a predictor for detecting device non-wear.

The present models deviate from the standard method of using consecutive zero counts as a proxy for identifying device non-wear. This method instead examines changes in device acceleration and device inclination simultaneously to more robustly detect periods of non-movement. In examining the model performance of the three models applied in this study, it is important to note that the three strongest predictors in the combined site model were all ankle predictors making the combined site model closely resemble the ankle only model. In fact, the ankle only model performed slightly better than the combined site model despite an equitable split in wear time for the hip and ankle devices. This is likely due to the fact that the hip only model contributed very little to the combined site model and in some cases resulted in a misclassification. This can likely be attributed to the mostly structured nature of the criterion data used in development. The infants were in similar positions during the wear periods meaning little hip movement occurred in most cases. One difference from the limited literature in this sample population that should be noted is the use of a hip-worn device compared to a wrist-worn device [7, 19]. Future studies focused solely on detecting independent infant movement could consider using wrist- and ankle-devices in a population this young as they are primarily non-locomotive except for caregiver generated movement or manual manipulation. However, for identifying waking time, the hip should provide valuable information about device wear and caregiver interaction since the device is affixed to underpants worn over the diaper and would need to be removed when changing whereas a device worn on the wrist or ankle would not need to be removed. Thus, it may be advantageous to pair a hip-worn device with either a wrist- or ankle-worn device for monitoring wear status and distinguishing movement characteristics.

In the two infant studies available in the literature, the identification of waking wear time is prioritized during data cleaning [18, 19]. Pitchford et al. [19] and Ketcheson et al. [18] posited that two or more minutes of consecutive zero counts would be representative of an infant that was not moving and therefore likely sleeping or the device was not being worn. Thus, the consecutive zeros method was developed to clean periods of non-wear, non-movement, and sleep leaving only active waking wear time. While the consecutive zero counts method was used on wrist and ankle data in the original studies, it is reasonable to believe that brief periods of movement to indicate waking time would also be captured at the hip in a similar fashion. Since the consecutive zero counts method was developed as a cleaning step to identify waking wear time, applying the methods to hip data seems acceptable in this case since the only goal of this step was to identify movement using counts.

In an effort to quantify the magnitude of misclassification that might occur from using count data to classify waking wear device wear time was compared against secondary criterion levels of sleeping and waking time. The consecutive zero counts method, which was developed to identify waking wear time, identified fewer minutes of waking wear time than the decision tree method developed in the present study. On average, the decision tree methods classified 95.9% of waking wear minutes correctly compared to 82.8% for the consecutive zero counts method. While true that extended periods of non-movement likely correspond to either sleep or non-wear time, it is difficult to disentangle the two from one another in free-living circumstances. With sleep being a predominant behavior in infants, removing extended periods of non-movement will likely include removing a large proportion of sleep time leaving little data remaining to work with. Ideally, periods of sleep are retained as part of the dataset and only true periods when the device is not being worn are removed. This demonstrates a principal drawback to using only movement data to classify device wear status demonstrating that using movement and position data simultaneously allows for more robust detection of device wear, even when factoring in periods of sleep.

A contributor to the performance increase in this study was the inclusion of device inclination. This information is supplementary to changes in device acceleration and seems to help create a more stable predictor of device wear in cases where little to no movement occurs. This can also be true for change in movement when the device is not being worn, is moving, and is in a position that may occur when it is being worn (e.g. the device is clipped to a diaper bag or car seat). However, the position is similar to a device that is being worn, leading to misclassification of the non-wear time as wear. This is a limitation, but one that currently exists with other methods as well. The present methods should at least provide more robust criteria for assessing these time periods and have greater potential for correctly classifying those data points. As device data are more robustly segmented, this area of misclassification should be further examined in future studies.

With the shift from counts to raw data there are several additional considerations that need to be evaluated when using raw data samples for predictive modeling. For example, the raw data signal can be transformed based on the data processing and reduction steps taken, including signal filtering and autocalibration. With this in mind, the decision was made to use change in device acceleration (CV) rather than the raw acceleration data features to create a more stable predictor of movement. This is in line with the findings of Ahmadi et al. [29] who suggest that using features focused on change in acceleration instead of the absolute acceleration values is more valuable for the discrimination of non-wear. This was due to the inherent intra- and inter- device variation in the raw acceleration signal making the acceleration features susceptible to misclassification simply based on the inherent differences in the sensor trial-to-trial. Additionally, one reason the single-axis acceleration and VM values were not considered as valuable of classifiers compared to the CV data is because of the bi-directional raw signal. With the sign of the acceleration magnitude being contingent on the deice attachment site and the device orientation, there was some confusion for the decision tree about what sign of the same magnitude acceleration actually corresponds to wear or non-wear. For example, if the non-wear threshold for the Y-axis at the hip is < -3 mg then -100 mg would be classified as non-wear. This orientation dependence makes the individual axes susceptible to this issue of no consensus about what data truly corresponds to wear and non-wear and that is another reason those data were excluded from the final analyses. This decision should make this method more generalizable and less prone to overfitting nuances in the acceleration signal. Additionally, the raw signal was used to compute inclination angles for all three sensor axes. This resulted in more stable features representing device inclination and change in movement in place of the raw sensor values themselves.

This study is not without limitations, among which are the development environment (i.e. laboratory conditions) and structured protocol (i.e. placement in an infant rocking chair) used to establish criterion labels for the majority of wear minutes. In comparison to the total volume of wear data collected, there was a limited amount of criterion labelled wear data outside of the infant rocking chair that did not include handling by a caregiver (i.e. infant generated movement). The present study uses a very young sample of participants making it challenging to draw many comparisons to the literature where study samples in this population are typically a little older which plays a role in development status and movement patterns. An additional limitation to this study includes a lack of validated measures for objectively assessing infant PA as accelerometer-based devices and inertial sensors have been used to monitor infant PA but have not been validated as an accurate measure of infant PA. The lack of device placement standardization is an additional limitation as the device placement between the right and left sides of the body results in different angles of inclination. However, no standardization of device placement does make the final models used for this study available to use with data from devices worn on either side of the body.

The choice to work with the raw sensor data (mg) in 1-minute windows could additionally be considered a limitation as this window length does average out some small movements that may detectable in shorter windows (e.g. 1-s) that would indicate movement and therefore device wear in most instances [30]. However, the resolution of the criterion measure of device wear available in this study was recorded to the nearest minute making it most appropriate to work the sensor data in 60-s windows. Migueles et al. [31] highlights that it is generally an acceptable practice to collapse raw acceleration data to 60-s windows as this window length is considered sufficient resolution for many PA assessment applications as it makes for more simplified data handling, analysis, and interpretation [32]. Trost et al. [33] has also noted previously that for classifying activity type in children, markedly greater agreement could be achieved when using a 60-s window length (88.4%) compared to using a shorter 10-s window length (81.3%) with machine learning methods. Nevertheless, the true impact of window size in this population is presently unknown and should be considered in future analyses. While true that valuable information pertaining to fine movements exists in the raw sensor signal and is useful for tasks such as activity classification, working with the raw signal is not always the most practical as it is susceptible to signal noise not due to movement as well as device calibration concerns. Furthermore, some applications do not benefit from using the raw sensor signal which can complicate analyses if the statistical method chosen cannot sufficiently leverage the information available in the signal [32]. The study methods presented are intended to quantify gross device wear and non-wear time as a primary data cleaning step. Once wear time has been identified, the raw sensor signal can be used to quantify end-points of interest (e.g. PA type) if desired, since the rich information in the signal may be better utilized in these applications if the criterion appropriately matches the sensor resolution chosen.

The length of non-wear periods in infants that is considered interesting and relevant has yet to be established. Infants at this age (1–3 months) typically eat every 2–3 hours with waking and sleeping periods in between. Presently, maintaining short periods for identifying wear and non-wear seems plausible due to infant behaviours and movement patterns. Previous research operated using 2-minute periods of non-movement to identify non-waking wear time and non-wear time [18, 19]. Lastly, the choice of a simple decision tree as the method for determining wear/non-wear may seem like an overstep in terms of the complexity but the motivation for the choice includes intentions of using this method as a building block to adapt and improve upon in future research where additional decisions can be added such as parsing infant movement from caregiver-generated movement. Nevertheless, a first step in evaluating monitor data requires adequate cleaning, and this method provides an automated approach to identify periods of wear and non-wear as a preliminary step in data cleaning.

This study has several strengths including presenting a novel approach for using raw sensor data to objectively determine device wear compared to current conventions. The methods present multiple models which increases applicability since some studies use only a single attachment site instead of multiple attachment sites. This study provides a necessary starting point for evaluating infant movement using wearable device data as movement patterns in infants are unique to the population and require special methods for assessment.

## Conclusions

In conclusion, the decision tree methods presented in this study provide an objective method for robustly discriminating device wear time from non-wear time in infants using both a combined site and independent site approach. Overall, this approach provides an easily implementable method with interpretable results. While the infant population used for development is unique, these methods should translate to developing a comparable model for youth and adults rather readily and will be the focus of future research. Understanding physical activity development and patterns across the life course can be possible with the use of wearable devices such as accelerometers and use of standardized data reduction and analytic methods to quantify wear and non-wear time reproducibly.

## Supporting information

S1 Dataset (CSV)
